# Pilot study of a compassion meditation intervention in chronic pain

**DOI:** 10.1186/s40639-014-0004-x

**Published:** 2014-10-27

**Authors:** Heather L Chapin, Beth D Darnall, Emma M Seppala, James R Doty, Jennifer M Hah, Sean C Mackey

**Affiliations:** 1 Department of Anesthesiology, Perioperative and Pain Medicine, Division of Pain Medicine, Systems Neuroscience and Pain Lab, Stanford University, 1070 Arastradero Road, Suite 200, MC 5596, Palo Alto, CA 94304, USA.; 2 The Center for Compassion and Altruism Research and Education, Stanford University, 1070 Arastradero Road, 2nd Floor, Palo Alto, CA 94304, USA.

**Keywords:** Compassion, Meditation, Anger, Chronic pain, Women

## Abstract

**Background:**

The emergence of anger as an important predictor of chronic pain outcomes suggests that treatments that target anger may be particularly useful within the context of chronic pain. Eastern traditions prescribe compassion cultivation to treat persistent anger. Compassion cultivation has been shown to influence emotional processing and reduce negativity bias in the contexts of emotional *and* physical discomfort, thus suggesting it may be beneficial as a dual treatment for pain and anger. Our objective was to conduct a pilot study of a 9-week group compassion cultivation intervention in chronic pain to examine its effect on pain severity, anger, pain acceptance and pain-related interference. We also aimed to describe observer ratings provided by patients’ significant others and secondary effects of the intervention.

**Methods:**

Pilot clinical trial with repeated measures design that included a within-subjects wait-list control period. Twelve chronic pain patients completed the intervention (F= 10). Data were collected from patients at enrollment, treatment baseline and post-treatment; participant significant others contributed data at the enrollment and post-treatment time points.

**Results:**

In this predominantly female sample, patients had significantly reduced pain severity and anger and increased pain acceptance at post-treatment compared to treatment baseline. Significant other qualitative data corroborated patient reports for reductions in pain severity and anger.

**Conclusions:**

Compassion meditation may be a useful adjunctive treatment for reducing pain severity and anger, and for increasing chronic pain acceptance. Patient reported reductions in anger were corroborated by their significant others. The significant other corroborations offer a novel contribution to the literature and highlight the observable emotional and behavioral changes in the patient participants that occurred following the compassion intervention. Future studies may further examine how anger reductions impact relationships with self and others within the context of chronic pain.

## Background

Chronic pain affects an astounding 100 million Americans - causing suffering for the individuals afflicted, their families, and significant others. Chronic pain also exerts a significant financial burden on the U.S. economy, costing over half a trillion dollars annually [1]. The relationship between pain and psychosocial factors suggests that pain involves dynamic interactions between the mind/brain, body, and environment [2,3]. As such, chronic pain can negatively impact a person’s cognitive and emotional state [4,5] as well as their relationship with themselves [6] and others [7–10]. Conversely, pain also may be inhibited or facilitated by various cognitive, emotional, and contextual factors that influence ascending and descending neural pathways [11–13], suggesting that effective cognitive and emotional interventions may positively influence these pathways and reduce pain.

Anger is an important emotional correlate of chronic pain [6,14–25], with studies showing that chronic pain is associated with anger and animosity towards others as well as towards oneself [6,19,23] including disappointment and frustration with pain [26], self-blame [27], self-criticism [28] and poor acceptance of one’s physical limitations [29]. Furthermore, anger is associated with reduced pain treatment response [9,30] and impaired relationships with spouses [10]. Accordingly, we investigated a mind-body intervention—compassion meditation- to address both pain and anger.

Compassion is the experience of perceiving suffering and wishing to alleviate that suffering. We selected compassion meditation because compassion – which this meditation is meant to train – is shown to increase psychological well-being and interpersonal relationships. Compassion is linked to less judgmental views of others [31], greater life satisfaction [32], decreased anxiety, depression, and chronic distress, and positive emotions as a consequence of compassionate acts [33]. Individuals with greater compassion experience better relationships and are regarded by others more positively [34]. Compassion meditation is prescribed as treatment for persistent anger in Eastern cultures [35].

Strengthening compassion is not a new concept in Eastern contemplative traditions, which have meditation practices devoted specifically to cultivating feelings of loving-kindness and compassion. Through the recognition of a common humanity, these practices involve the development of feelings of love and compassion that are directed to the self, others, and ultimately towards all beings [36]. Despite the rich history of these established practices in contemplative traditions, compassion training is a relatively new area of study in the Western world [37] (review).

Compassion meditation is based on broader, general meditation practices, which have been shown to be effective for reducing a wide variety of negative cognitive and emotional state–and psychopathology such as anxiety and depression [38,39]. Meditation-based interventions have few if any side effects [40,41], are cost- and time-effective [41–43], and provide patients – who learn tools to help themselves - with a sense of autonomy [43] and self-mastery [44].

In compassion meditation training participants learn to experience feelings of compassion while meditating on various relationships (e.g., self, partner, difficult people, strangers). Accordingly, the intention of compassion meditation training is to increase a person’s capacity to experience compassion for themselves, others, and the world [45]. Compassion may be cultivated through training [46–48] and has longitudinal effects, with gains in compassion maintained at one year follow-up [47]. Compassion-focused therapeutic interventions [49,50] have been shown to reduce depressive symptoms in various populations [51–53]. Compassion interventions are also associated with increased positive emotions [54], feelings of social connectedness [55], pro-social behavior [56] and improved health outcomes in the form of optimized immune and stress responses [57]. Within the context of chronic pain, self-compassion has been associated with greater chronic pain adaptation and acceptance [58]. Because of its effect on decreasing anger and increasing self-compassion and interpersonal compassion, compassion meditation may particularly benefit for individuals with chronic pain.

Indeed, prior work on interpersonal relationships has shown heightened distress and marital dissatisfaction in the spouses of people with chronic pain [7]. The marital dissatisfaction appears to be partially mediated by the level of emotional distress of the person with pain [7]. Of particular interest is the finding that anger and hostility in individuals with chronic pain predicts depression and marital satisfaction in their spouses [10]. Furthermore, anger in individuals with chronic pain also has specific discriminative value: studies of emotion profiles have shown that anger distinguishes healthy individuals from those with chronic pain and further discriminates between *depressed* individuals and those with chronic pain [59]. As such, interventions that specifically target anger may hold value for treating chronic pain, distress related to pain, and distress in the spouses and significant others of those living with chronic pain.

We found only one study that examined the effect of a compassion-based intervention – loving-kindness meditation training—on chronic pain [24]. In this study individuals with chronic low back pain were randomized to either standard care or to the 8-week compassion-based meditation program that was specifically adapted for chronic pain through inclusion of a chronic pain didactic component. Compared to the usual care group, participants who received the intervention had post-treatment reductions in pain, distress, anger, and tension. Of interest, same-day pain and next-day anger were negatively correlated with the amount of time spent practicing loving-kindness meditation, suggesting a dose-dependent relationship with the meditation practice. Overall, the study provided evidence that a compassion-based meditation may improve pain and anger in individuals with chronic pain; however, the inclusion of the pain didactic component in the intervention introduced a confound that precluded clear understanding of the unique effect of the compassion-based meditation on pain and anger. Furthermore, we found no study to date to the effect of a compassion intervention on chronic pain acceptance.

The purpose of this pilot study was therefore to investigate whether a general compassion cultivation intervention—devoid of pain didactics – would improve pain, anger, and pain acceptance in individuals with chronic pain, and to test whether improvements would be corroborated by the 3rd person observation of their significant others. Our primary hypothesis was that compassion cultivation in individuals with chronic pain would result in reduced pain intensity and anger. We also hypothesized that the intervention would associate with reduced pain-related interference in various life domains and increased pain acceptance. Finally we hypothesized that reductions in anger and pain interference in individuals with chronic pain^a^ would be corroborated by significant other report [60].

## Methods

**Figure 1:**
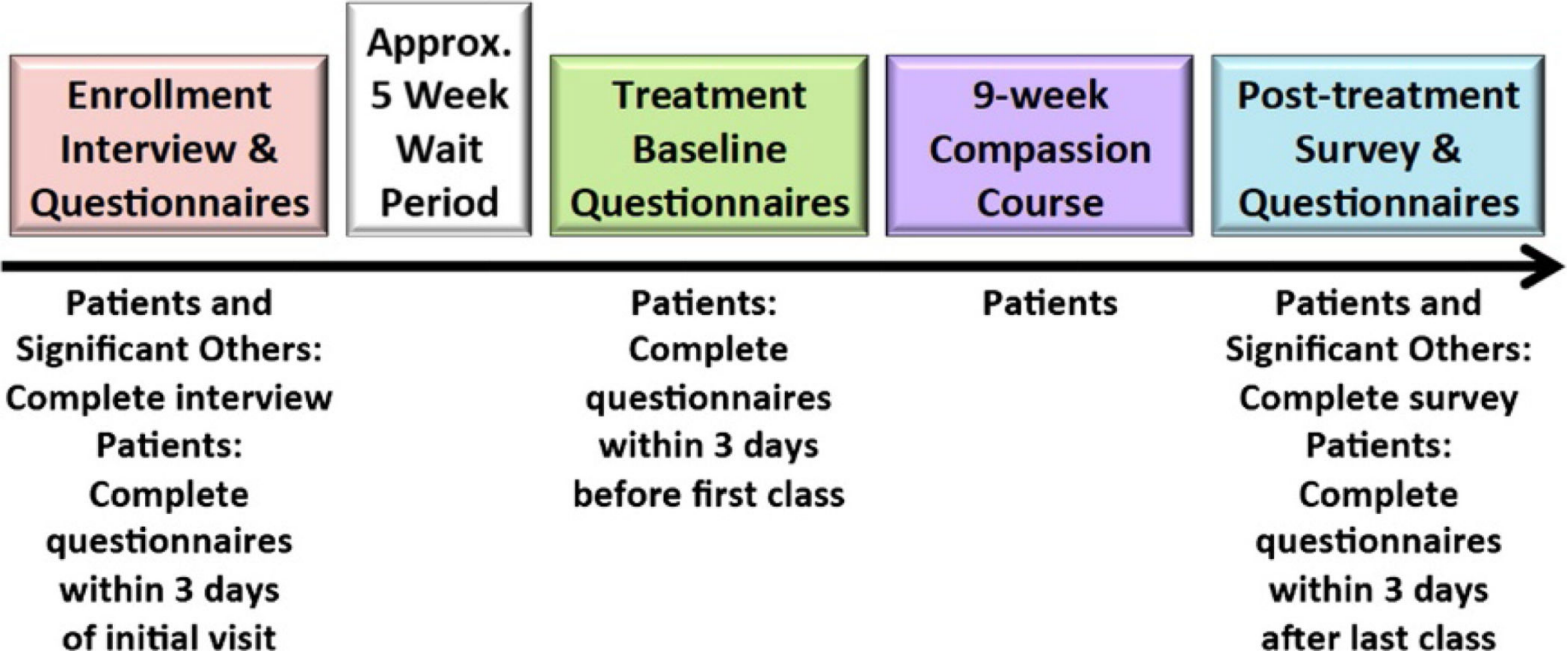
Study timeline.

In this pilot study we used a within-subjects, wait-list control with repeated measures design to characterize the effects of a compassion meditation intervention on chronic pain patients and their significant others. A link to an online questionnaire was emailed to all patients at three time points: at enrollment, after a 5-week post-enrollment waiting period (treatment baseline), and again after completion of the 9-week compassion training course (post-treatment); see Figure 1 for an illustration of the study timeline. Throughout the duration of the compassion cultivation intervention patients completed an online daily compassion meditation log to quantify the minutes they spent meditating that day.

During the 5-week waiting period, patients were instructed to simply “live their lives as they normally would”. This design allowed patients to serve as their own wait-list controls. Lack of significant differences between enrollment and treatment baseline time points would lend confidence that any observed post-treatment changes would be attributable to the compassion intervention rather than to time effects (e.g., regression to the mean). To produce a rich and maximally informative dataset, we utilized a mixed-methods approach including both quantitative questionnaire data and qualitative interview and survey data.

Interviews were conducted in-person at the enrollment visit and surveys were administered online at treatment baseline and post-treatment following the last compassion class (described below). The patients’ significant others completed interviews at the same time points as the chronic pain patients. Significant others were not allowed to attend the compassion cultivation intervention because we were specifically interested in the observer reports of the significant others, and in any secondary effects of the compassion intervention. The study was approved by the Stanford University Institutional Review Board (IRB). All patients and patients’ significant others provided written, informed consent as approved by the IRB.

### Chronic pain patient participants

**Figure 2:**
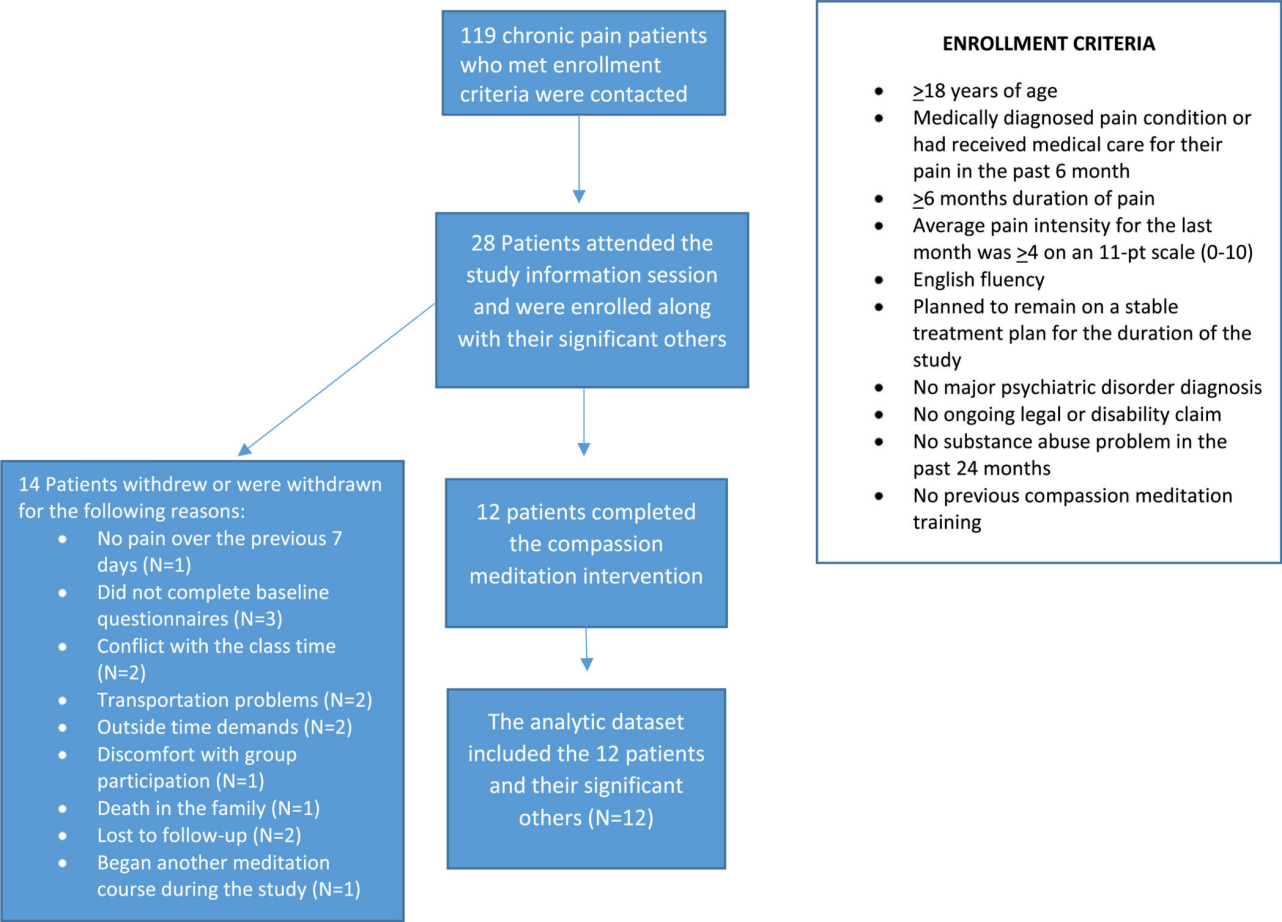
Participant flow chart.

Patients with chronic pain (N = 119) were contacted from a database of individuals with chronic pain that had previously expressed interest in research participation at the Stanford Systems Neuroscience and Pain Lab. Figure 2 displays the participant flow chart and describes the inclusionary and exclusionary criteria for the study. Twenty-eight chronic pain patients attended the information session and were enrolled in the study (24 female, mean age = 49.61, SD = 10.59). Figure 2 describes the reasons for participant drop out or withdrawal from the study (N = 14). The twelve patients who completed the study were included in the analytic dataset (F = 10; mean age = 48.33, SD = 10.80) (See Table 1).

### Significant other participants

Patients were asked to identify a significant other to participate with them in the study – someone with whom they had a close relationship and interacted with frequently (e.g., spouse or life partner, girlfriend or boyfriend, family member, or close friend). Patients then asked their significant others to contact the lab to be screened over the phone and schedule a time to attend an initial information session with their chronic pain study partner. Significant others were included if they were 18 years of age or older and excluded if they were diagnosed with a major psychiatric disorder, reported having a substance abuse problem in past 24 months, or had prior compassion meditation training. All significant others of the enrolled patients were eligible and provided informed consent (N = 28, 7 female, mean age = 50.71, SD = 12.59). Only the significant others of patients who were retained in the study were included in the final analysis (N = 12; 3 female, mean age = 49.17, SD = 11.48). Significant others’ demographics and relationships to the patients are shown in Table 2.

The significant others did not receive the compassion cultivation intervention. Rather, the role of the patients’ significant others was to provide pre- and post-treatment observer information about their chronic pain patient partners.

### Compensation

All enrolled chronic pain patients and their significant others each received $35 at the initial information session and were mailed $50 checks after completion of the last battery of questionnaires and final post-course survey questions. In addition, the chronic pain patients received the Compassion Cultivation Training (CCT) course at no cost to them.

### Compassion cultivation training course

Chronic pain patients attended a standardized 9-week CCT course developed by Stanford’s Center for Compassion and Altruism Research and Education (CCARE). The intervention was delivered to all patients simultaneously in a single class cohort. An experienced CCT instructor who was certified through CCARE’s instructor training program taught the course. To ensure that no pain didactics were delivered during the class, the instructor selected for the study had no formal pain training or background delivering the intervention to patients with chronic pain.

The course involved weekly 2-hour class meetings that included didactics specific to compassion meditation practices, in-class meditation practice, and small and large group discussions. Patients were also given a CD containing all of the guided meditations. A class website provided an optional forum for patients to discuss their experiences involving their in-class or at-home practice. The instructor posted on the website audio recordings of meditations that were themed for the week and any materials that were discussed in class. The website was available only to the patients and no data were collected from their in-class or online discussions. Significant others were *not* allowed to attend the course with the patients, and patients were asked to not share with their significant other any material from the class (e.g., audio recordings of guided meditations).

As a foundation for compassion meditation training, the first 2 classes involved basic instruction in mindfulness meditation. The compassion meditation training began in week 3 with a practice centered on cultivating feelings of compassion for someone with whom they had a close relationship. Weeks 4 and 5 were devoted to developing self-compassion, week 6 to developing compassion for strangers, and week 7 focused on cultivating compassion for difficult people through the recognition of a common humanity. In week 8, chronic pain patients were taught a “Tonglen” meditation practice, in which they learned to imagine taking in suffering (either in general or from a specific person) on the in-breath and release suffering on the out-breath. The class closed in week 9 with suggestions on how to continue to integrate compassion practices into their daily lives. Over the 9-week period, patients were asked to keep a daily online record tracking the number of minutes of meditation they practiced. Patients were provided additional, optional homework exercises related to the compassion theme each week (e.g. writing a compassionate letter to oneself).

### Variable measurement

#### Chronic pain patients

##### Anger

The Patient Reported Outcomes Measurement Information System (PROMIS) Anger Scale [short form, 61] is a validated measure [62] that includes 8 items for which respondents rate their frequency of angry feelings and reactions experienced over the previous 2 weeks using a 5-point scale (e.g., I was irritated more than people knew, 1 = never, 5 = always). PROMIS Anger has been used in chronic pain research [63].

##### Pain severity

All pain-specific measures are validated and widely used in chronic pain research. Pain severity was calculated as an average of four questions of the Brief Pain Inventory short form [BPI, 63] in which respondents rate their worst, least and average pain over the past week, as well as their current pain severity. All items use an 11-point response scale (0 = no pain, 10 = pain as bad as can imagine).

##### Pain-related functional interference

Pain Interference was also measured by the Brief Pain Inventory Short Form [64]. Seven items assess to what degree pain interferes with functioning in 7 life domains (general activity, mood, walking ability, normal work, relations with other people, sleep, and enjoyment of life) on an 11-point scale (0 = does not interfere, 10 = completely interferes). The ratings for the 7 items were averaged to create a composite pain-related functional interference score.

### Chronic pain acceptance

Pain acceptance was measured with the Chronic Pain Acceptance Questionnaire [58], a 20-item self-report questionnaire in which respondents use 7-point scale (0 = never true, 6 = always true). Sample item: “I am getting on with the business of living no matter what my level of pain is.” Higher scores are indicative of greater pain acceptance and adaptive adjustment to chronic pain.

Patients and significant others were asked to refrain from discussing their survey responses with one another. All questionnaires were presented in random order for all patients. Questionnaires were distributed and completed through the Stanford University online survey system (Qualtrics, Provo, UT, http://www.qualtrics.com/) which meets the Health Insurance Portability and Accountability Act (HIPAA) compliance standards.

### Semi-structured interviews/surveys

#### Enrollment chronic pain patient interview

Patients and Significant Others completed an in-person, semi-structured interview with study staff at study enrollment. The interview contained quantitative and open-ended questions. The purpose of the enrollment interview was to collect information about patients’ past meditation experience and expectations about the study. Open-ended questions asked about their past meditation experience and any current meditation practices. Only specific experience with compassion meditation was exclusionary for the study. In terms of treatment expectations, patients were asked how much they expected the compassion intervention would improve their pain and quality of life on a 0–10 scale (0 = no improvement, 10 = complete improvement.)

#### Enrollment significant other interview

The significant others were asked to rate how much they expected the course would improve their partner’s pain (0 = no improvement, 10 = complete improvement) and their partner’s quality of life (0 = no improvement, 10 = complete improvement). Open-ended questions asked significant others about their past meditation experience and any current meditation practices.

### Post-treatment online survey

#### Patient post-treatment online survey

Immediately following the last class, all patients were emailed a link to the online post-treatment survey that consisted of both quantitative and open-ended questions. Patients were asked to rate on a 0–10 scale how much they thought the course improved their pain and quality of life (0 = no improvement, 10 = completely improved). If they rated > 0 improvement, patients were then asked open-endedly which aspects of their quality of life they felt were changed as a result of the compassion intervention. Additionally, patients were asked whether they thought the intervention changed the way they relate to themselves, to their significant others, and to the outside world, and if so, to provide examples.

#### Significant other post-treatment online survey

Immediately after their partner completed the compassion intervention, each significant other received a link to the post-treatment survey. Significant others were asked to rate how much they thought the course improved their chronic pain partner’s pain and quality of life (0 = no improvement, 10 = completely improved). If they rated > 0 improvement, they were asked open-endedly which aspects of their partner’s life were improved by their chronic pain partner having participated in the compassion intervention. Finally, they were asked whether they observed changes in the way their patient partner related to themselves, to them (the significant other), or to the outside world, and if so, to provide examples.

### Analysis

One-way repeated measures analysis of variance (RM-ANOVA) tests were conducted for each questionnaire using SAS Version 9.4 (SAS Institute Inc., Cary NC) with time as the repeated measures factor. Where significant main effects were found, post hoc paired t-tests were calculated to determine which time points differed significantly (e.g., pre- compared to post-course time points). The primary outcome measures were BPI Pain Severity and PROMIS Anger, with BPI Pain Interference and Pain Acceptance as secondary outcomes. Pearson correlation was used to examine relationships between time spent in compassion meditation and the change in score from pre-course to post-course of the primary and secondary outcomes. In regression analyses, time spent in compassion meditation was examined as a predictor (process) variable. Secondary outcomes were corrected for multiple comparisons using a False Discovery Rate correction (corrected minimum p value needed for significance = .024). All other measures were exploratory and were not corrected for multiple comparisons given the small sample size. Technical difficulties prevented the Pain Acceptance questionnaire from displaying properly in the online system at baseline for half of the patients. Therefore, a paired t-test was conducted to determine changes in pre- to post-course Pain Acceptance because data was available for all 12 patients at both of these time points.

## Results

### Chronic pain patients

No differences were found between participants who completed the study and those who withdrew or were withdrawn in terms of our primary measures (Pain Severity, Pain Interference, or anger).

#### Questionnaires

**Figure 3:**
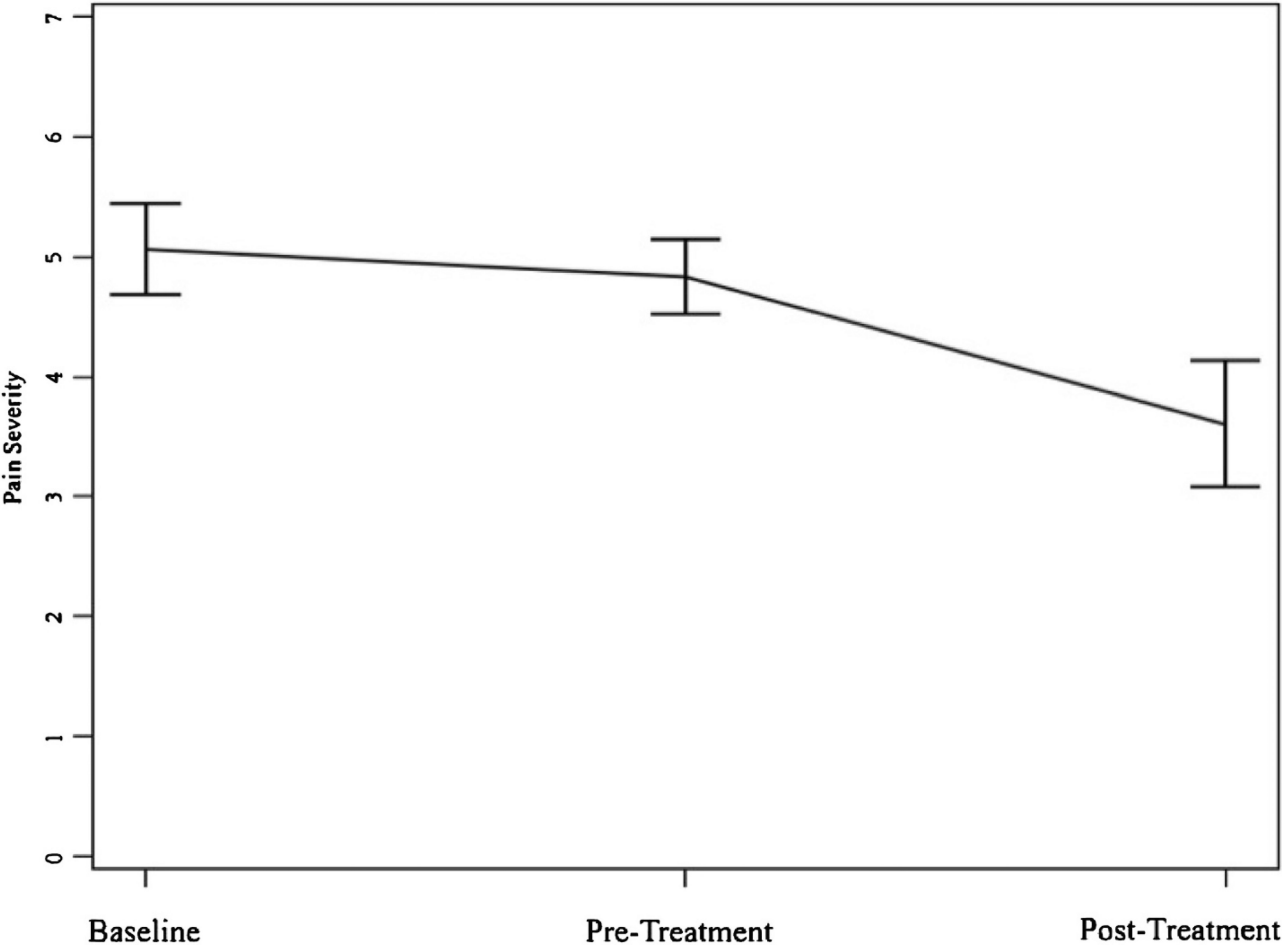
Pain severity across time.

**Figure 4:**
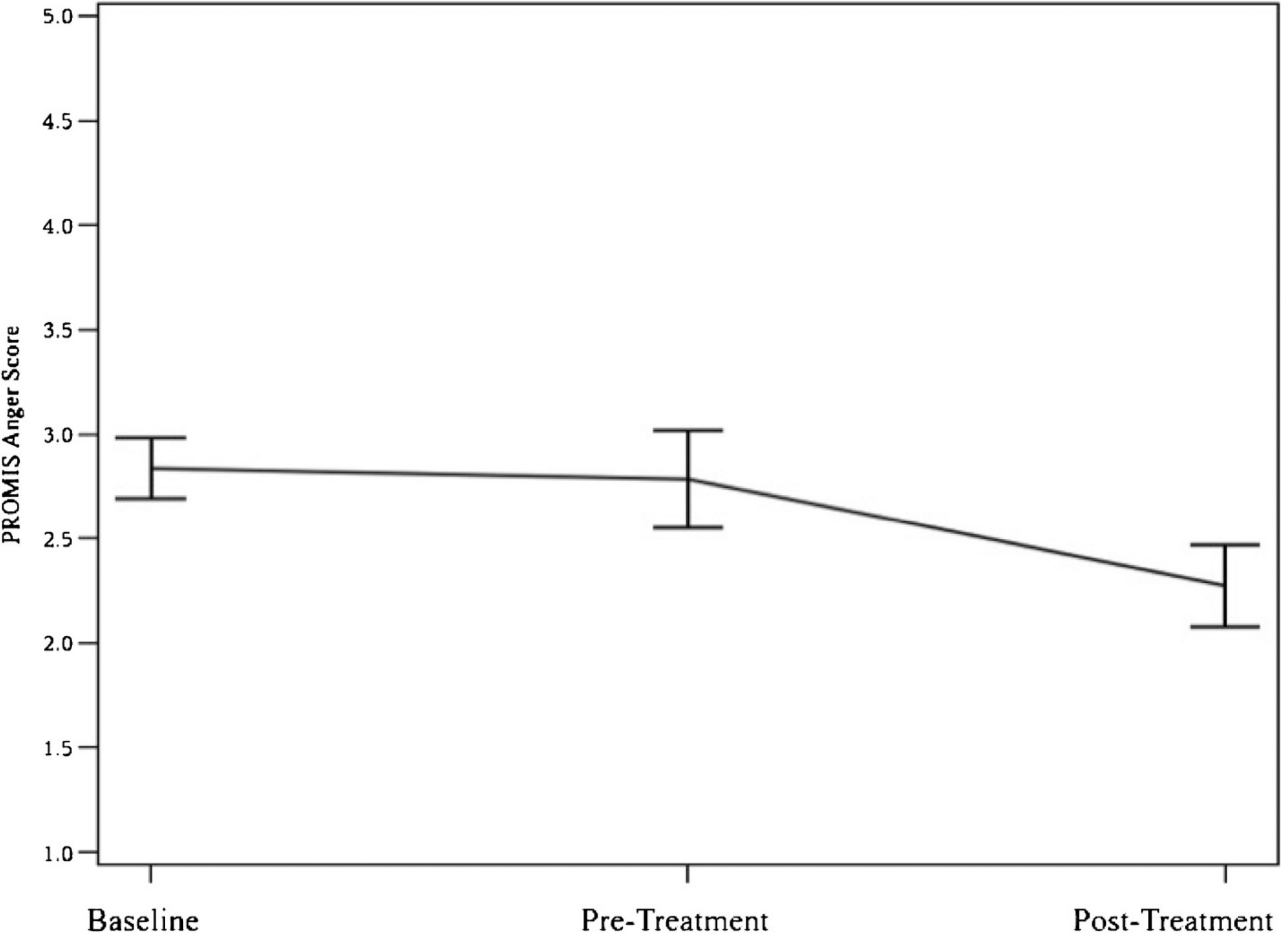
Anger across time.

Table 3 displays the results for each measure across the study time points. As expected, we found no significant differences between enrollment and treatment baseline ratings for any of the primary measures (BPI Pain Severity, PROMIS Anger score) with post-hoc paired t-tests. As such, the remainder of the results describe the RM ANOVA results and subsequent contrast between the pre- and post-treatment time points. As hypothesized, a significant difference existed across all time points for BPI pain severity (Table 3). BPI Pain Severity was significantly reduced at post-treatment compared to enrollment and treatment baseline; t(11) = 3.75, p = 0.003 and t(11) = 2.45, p = 0.03 respectively (See Figure 3). Similarly, a significant difference existed across all time points for PROMIS Anger score (Table 3). PROMIS Anger score significantly decreased at post-treatment compared to enrollment and treatment baseline; t(11) = 2.67, p = 0.02 and t(11) = 2.92, p = 0.01 respectively (See Figure 4). Additionally, paired t-tests showed significant improvements for Pain Acceptance pre- to post-treatment; t(11) = −2.94, p = 0.01).

Change scores were calculated for the primary and secondary outcomes by subtracting pre-treatment scores from post-treatment scores. Subsequently, Pearson product–moment correlation coefficients were computed to assess the relationship between total minutes spent in compassion meditation and the change scores (See Table 4). A positive correlation between time spent in compassion meditation and change in pain acceptance score did not reach significance (r = 0.48, p = 0.12), nor did any of the other correlations tested.

#### Patient enrollment interview

During the enrollment interview, 50% (N = 6) of chronic pain patients reported having some past meditation experience (ranging from listening to meditation podcasts to attending a meditation course or retreat), 42% (N = 5) reported having other experiences they considered to be related to meditation (e.g., praying, biofeedback, breathing exercises), and 8% (N = 1) reported having no previous meditation experience. No patients reported having a consistent formal meditation practice at the time of the interview. For the treatment expectancy measure, patients reported moderate expectations for improvement in pain (M = 5.21, SD = 1.57) and quality of life (M = 6.96, SD = 1.33). Expectations for improvement in pain were significantly correlated with the change in pain acceptance score (r = 0.66, p = 0.02) and the change in PROMIS Anger score (r = −0.63, p = 0.03). In addition, expectations for improvement in quality of life were correlated with the change in PROMIS Anger score (r = −0.68, p = 0.02) (See Table 4).

#### Patient post-treatment online survey

Patients reported that the course improved their pain to a moderate degree (M = 4.58, SD = 3.62) and quality of life to a similarly moderate degree (M = 6.58, SD = 1.98). Patient responses to open-ended post-treatment survey are summarized in Table 2.

### Patients’ significant others

#### Enrollment interview

Fifty percent (N = 6) of patients’ significant others reported having some past meditation experience (ranging from meditating at the end of yoga classes to studying with Tibetan and Japanese Buddhist priests for 3 years). Similar to the patients, on average the significant others had moderate treatment expectations for their chronic pain patient partners for improvement in pain (M = 4.38, SD = 2.35) and quality of life (M = 5.88, SD = 1.93).

#### Post-treatment online survey

Overall the significant others observed moderate improvement in their partner’s pain (M = 4.72, SD = 2.93) and quality of life (M = 5.08, SD = 2.60) post-treatment. The significant others’ ratings for post-treatment improved quality of life in their chronic pain partner was correlated with the change in PROMIS Anger score (r = .68, n = 12, p = .016). No other correlations between significant other’s ratings for post-treatment improved quality of life in their chronic pain partner and outcomes were significant.

Our results indicated that greater change in anger for the chronic pain patients’ correlated with significant others’ ratings for post-treatment improved quality of life in their chronic pain partners (r = .68, n = 12, p = .016).

## Discussion

In chronic pain, anger has been shown to have negative effects on pain severity [15,16], pain treatment response [9,30], quality of life, and on relationships with others [10,20]. Studies have also shown that the spouses and partners of people with chronic pain are more negatively impacted if the person with pain has concomitant anger [10,20]. The main goals of this study were to determine the preliminary effect of a compassion meditation intervention for reducing pain severity and anger in patients with chronic pain and to describe third person observations made by their significant others. Our patient sample was predominantly female, and therefore at this stage our results only inform the effect of the compassion meditation intervention in women.

The study yielded some unique preliminary findings. First, we found that the compassion intervention was associated with post-treatment reductions in pain severity for chronic pain patients that were moderate in effect size and clinically important in magnitude [65]. Our small sample allows for preliminary evidence only; however, the magnitude of the effect of the intervention on pain intensity is similar or greater than those reported for studies of cognitive behavioral therapy [66].

We also found that the compassion intervention was associated with a 25% reduction in anger (p = 0.01); IMMPACT guidelines for clinical importance classify this level of reduction as important but in the minimal range [67]. It is possible that a larger sample – enriched for high anger at baseline – may evidence greater effects. Given that people with chronic pain often experience frustration with their bodies and therefore themselves, compassion meditation may directly treat this psychological difficulty.

We found that people who underwent the compassion intervention had increased pain acceptance at post-treatment, and highlight this as a variable for consideration in larger studies that may allow for modeling and mediational analyses.

Unlike prior compassion intervention research [24], the CCARE Compassion Cultivation Training was not tailored for chronic pain. Rather, the course was developed for the general public, and as such, contained no pain didactics nor any specific focus on attention to somatic awareness. Nevertheless, we observed significant changes in pain severity, anger, and pain acceptance in patients with chronic pain. Thus, our preliminary findings build on the prior work of Carson and colleagues [24], and suggest that a compassion meditation intervention confers unique benefit in the chronic pain context.

Patient reported reductions in anger at post-treatment were qualitatively corroborated by several of the significant other statements. While none of the significant other questions specifically asked about anger, it emerged as a major theme with significant others offering unsolicited descriptions about anger in their chronic pain partners in the open ended interview/survey. For example, several significant others mentioned the anger of the chronic pain patients in the baseline interviews (e.g., “…she tends to become angry more easily…,” and “She’s got sort of a short fuse as far as some anger issues.”). In the post-treatment survey several significant others commented on anger reductions in their chronic pain partners (e.g., “…she seems to recover from bouts of anger much more quickly than before”. “She is less likely to “go off the handle”. “Less incidence of anger flashes”. One significant other even noted a connection between the chronic pain patient’s anger and pain, “It was comforting knowing that she was getting compassion training, as I…have always believed that much of her pain is caused by anger”. These unsolicited observer reports further support the patients’ self-reported anger reductions and therefore indicate observable behavior change. Future studies may examine these associations in greater detail using quantitative measures for partner anger.

We hypothesized that the compassion intervention would have positive effect on pain related interference across life domains. However, our findings did not confirm significant effects. As such, it is unknown whether our sample size limited detection of potential effects, or whether the intervention simply does not impact these domains.

We did not find correlation between the time spent meditating and the change in anger, and this lack of association stands in contrast to prior work [24]. Possibly the benefits of the intervention are either largely achieved in the class itself, or in the mindful application of information learned in daily life, versus the time practicing compassion meditation. Alternatively, it is possible that a larger sample would replicate previous findings. Clearly, more research is needed to determine the specific pathways by which treatment response occurs. However, we found preliminary evidence to support our hypothesis that the compassion intervention would have positive impacts that could be observed by significant others. Namely, the correlation between change in PROMIS Anger scores and significant other’s ratings for post-treatment improved quality of life.

Taken together, our preliminary findings suggest multiple potential benefits associated with a general compassion intervention, including significant other reports of associated increased quality of life, reduced anger and pain severity in patients with chronic pain, and increased pain acceptance. Findings from this pilot study suggest that compassion cultivation may be a promising new intervention or adjunct to current treatment. Our results provided novel evidence that compassion meditation alone – in the absence of any pain management education or instructions – benefits people with chronic pain.

A strength of the study was that it included significant other observer ratings for pain severity. Post-treatment changes in pain severity reported by chronic pain patients were corroborated in direction and magnitude by their significant others in the quantitative and qualitative data. For the qualitative data, the concordance of themes that emerged between chronic pain and patients’ significant others provided an additional marker of validity for reported effects. Indeed, observations of pain reduction emerged as a theme in the qualitative data as evidenced in several of the significant others’ answers to the open-ended questions on the post-course survey (e.g., “…having a tool to help her relax I think has helped to prevent her pain from getting out of control…,” “She has been in less pain toward the end of the program,” and “There are more spans of calm, and pain free, anxiety free time.”). As stated earlier, the spontaneous observer reports for reduction in anger lend strength to the associations detected in the patient quantitative data.

Another strength of the study was the repeated measures design, with patients serving as their own wait-list controls. A clear lack of change in our variables of interest over the wait period (baseline compared to pre-course ratings) coupled with significant changes observed after the intervention (baseline compared to post-course ratings) lend confidence that the post-course changes were related to the intervention rather than regression to the mean.

Several limitations merit consideration. First, this was a small pilot study and thus we were likely underpowered to detect some effects that might emerge in a larger study. Another limitation was the lack of an active control group. We were therefore not able to distinguish the effects of compassion intervention from non-specific effects, such as positive effects of social interaction, social support or learning a new skill. For greater generalizability, future studies will also benefit from a larger and more representative sample size with a more balanced gender and ethnic distribution among patients and their significant others. Our relatively high attrition (50%) may be related to the unique challenges faced by people with chronic pain. Compared to those with other disorders (e.g., anxiety), patients with chronic pain have been shown to be less likely to complete similar 8-week meditation programs [68]. We found no differences between study completers and those who withdrew or withdrawn in terms of the baseline primary measures. The varied reasons provided for attrition preclude a single solution for retaining patients in a study that required such extensive time and travel commitments. Perhaps creating an online compassion training program that those with chronic pain can participate in from home may help alleviate some of the burden though this would lessen any potential social benefits to be gained by physically attending the class with others.

## Conclusions

Chronic pain imparts substantial psychosocial burdens to the individuals and their loved ones, thus underscoring the need to develop treatments that address these factors. Results from this pilot study provide preliminary evidence that a compassion cultivation intervention may reduce pain severity and anger and increase pain acceptance in patients with chronic pain. Despite the rich history of these established practices in contemplative traditions, compassion training is a relatively new area of study in the Western world [24,47,48,50–57,69–75]. More research is needed to determine which components of compassion training are most helpful, how much training is sufficient to effect change, for whom the training is most effective, the neural mechanisms that mediate positive changes, and the durability of clinical benefits. While compassion research is in its infancy [24,52], our findings hold promise for its role as an adjunctive treatment for people with chronic pain.

## Figures and Tables

**Table 1:** Chronic pain patient demographics

ID	Gender	Age	Ethnicity	Diagnosis/Pain location	Pain duration (Years)
1	F	52	Caucasian	Low back migraines	30.0
2	F	57	Caucasian	Low back	1.0
3	F	36	African American	Low back, neck, shoulder	2.0
4	M	54	Asian	Low back	0.5
5	F	55	Caucasian	Low back, widespread pain (fibromyalgia)	13.0
6	M	38	Asian	Low back	15.0
7	F	38	Hispanic	Low back, feet	15.0
8	F	61	African American	Low back, knee	20.0
9	F	50	Caucasian	Low back	9.0
10	F	29	Caucasian	Back	22.0
11	F	44	Other	Shoulder	10.0
12	F	66	Caucasian	Back	22.0

**Table 2:** Significant other demographics & relationship to patient

ID	Gender	Age	Ethnicity	Relationship to patient
1	M	66	Caucasian	Husband
2	M	58	Caucasian	Husband
3	M	35	African American	Husband
4	F	50	Asian	Wife
5	M	55	Caucasian	Husband
6	F	35	Asian	Wife
7	M	42	Caucasian	Domestic partner
8	M	56	African American	Close friend
9	M	45	Caucasian	Husband
10	M	31	Caucasian	Boyfriend
11	F	52	American Indian/Alaska Native	Sister
12	M	68	Caucasian	Husband

**Table 3:** Participant variables across study time points

Construct	Measure	MEANS(SE)			F Value	P-value
		Baseline	Pre-treatment	Post-treatment		
**Pain**	BPI Pain Severity (0–10)	5.06(0.37)	4.83(0.31)	3.60(0.53)	7.70	0.003
	BPI Pain Interference (0–10)	5.11(0.64)	4.42(0.53)	3.92(0.67)	2.54	0.102
	Pain Acceptance*	-	2.90(0.21)	3.56(0.22)	−2.94*	0.014*
**Anger**	PROMIS Anger (1–5)	2.84(0.15)	2.78(0.23)	2.27(0.20)	5.20	0.014

* A paired t-test was conducted for this variable.

**Table 4:** Correlation between total minutes spent in compassion meditation, expectations for improvement, and change scores*

Variable	Total minutes spent in compassion meditation	Expectations for pain improvement	Expectations for quality of life improvement
Change in BPI pain severity	−0.15(0.64)	0.11(0.73)	−0.33(0.30)
Change in BPI pain interference	−0.26(0.42)	0.19(0.56)	−0.22(0.49)
Change in pain acceptance	0.48(0.12)	0.66(0.02)§	0.48(0.12)
Change in PROMIS anger	−0.04(0.90)	−0.63(0.03)§	−0.68(0.02)§

* Results reported as Pearson product–moment correlation coefficient (*p*-*value*).

§ Significant correlation (*p*-value <0.05).

## References

[1] IOM: Relieving Pain In America: A Blueprint For Transforming Prevention, Care, Education, And Research (2011). Institute of Medicine Report.

[2] Melzack R, Wall PD (1965). Pain Mechanisms - a new theory. Science.

[3] Melzack R (1999). From the gate to the neuromatrix. Pain.

[4] McCracken LM, Iverson GL (2001). Predicting complaints of impaired cognitive functioning in patients with chronic pain. J Pain Symptom Manag.

[5] McWilliams LA, Cox BJ, Enns MW (2003). Mood and anxiety disorders associated with chronic pain: an examination in a nationally representative sample. Pain.

[6] Okifuji A, Turk DC, Curran SL (1999). Anger in chronic pain: investigations of anger targets and intensity. J Psychosom Res.

[7] Flor H, Turk DC, Scholz OB (1987). Impact of chronic pain on the spouse: marital, emotional and physical consequences. J Psychosom Res.

[8] Ahern DK, Adams AE, Follick MJ (1985). Emotional and marital disturbance in spouses of chronic low back pain patients. Clin J Pain.

[9] Burns JW, Higdon LJ, Mullen JT, Lansky D, Wei JM (1999). Relationships among patient hostility, anger expression, depression, and the working alliance in a work hardening program. Ann Behav Med.

[10] Schwartz L, Slater MA, Birchler GR, Atkinson JH (1991). Depression in spouses of chronic pain patients: the role of patient pain and anger, and marital satisfaction. Pain.

[11] Sud R, Ignatowski TA, Lo CPK, Spengler RN (2007). Uncovering molecular elements of brain-body communication during development and treatment of neuropathic pain. Brain Behav Immun.

[12] Fields HL (2000). Pain modulation: expectation, opioid analgesia and virtual pain. Biol Basis Mind Body Interact.

[13] Gatchel RJ, Peng YB, Peters ML, Fuchs PN, Turk DC (2007). The biopsychosocial approach to chronic pain: scientific advances and future directions. Psychol Bull.

[14] Conant LL (1998). Psycholgocial variables associate with pain perceptions among individuals with chronic spinal cord injury pain. J Clin Psychol Med Settings.

[15] Bruehl S, Chung OY, Burns JW (2003). Differential effects of expressive anger regulation on chronic pain intensity in CRPS and non-CRPS limb pain patients. Pain.

[16] Bruehl S, Burns JW, Chung OY, Ward P, Johnson B (2002). Anger and pain sensitivity in chronic low back pain patients and pain-free controls: the role of endogenous opiods. Pain.

[17] Kerns RD, Rosenberg R, Jacob MC (1994). Anger expression and chronic pain. J Behav Med.

[18] Gaskin ME, Greene AF, Robinson ME, Greisser ME (1992). Negative affect and the experience of chronic pain. J Psychosom Res.

[19] Hatch JP, Schoenfeld LS, Boutros NN, Seleshi E, Moore MA, Cyr-Provost M (1991). Anger and hostility in tension-type headache. Headache.

[20] Burns JW, Johnson BJ, Mahoney N, Devine J, Pawl R (1996). Anger management style, hostility and spouse responses: gender differences in predictors of adjustment among chronic pain patients. Pain.

[21] Sela RA, Bruera E, Conner-Spady B, Cumming C, Walker C (2002). Sensory and affective dimensions of advanced cancer pain. Psychooncology.

[22] Fernandez E, Turk DC (1995). The scope and significance of anger in the experience of chronic pain. Pain.

[23] Greenwood K, Thurston R, Rumble M, Waters SJ, Keefe FJ (2003). Anger and persitent pain: current status and future directions. Pain.

[24] Carson JW, Keefe FJ, Lynch TR, Carson KM, Goli V, Fras AM, Thorp SR (2005). Loving-kindness meditation for chronic low back pain: results from a pilot trial. J Holist Nurs.

[25] Carson JW, Keefe FJ, Goli V, Fras AM, Lynch TR, Thorp SR, Buechler JL (2005). Forgiveness and chronic low back pain: a preliminary study examining the relationship of forgiveness to pain, anger, and psychological distress. J Pain.

[26] Dow CM, Roche PA, Ziebland S (2012). Talk of frustration in the narratives of people with chronic pain. Chronic Illn.

[27] Cedraschi C, Girard E, Luthy C, Kossovsky M, Desmeules J, Allaz AF (2013). Primary attribution in women suffering from fibromyalgia emphasize the perception of a disruptive onset for a long-lasting pain problem. J Psychosom Res.

[28] Rudich Z, Lerman SF, Gurevich B, Weksler N, Shahar G (2008). Patients’ self-criticism is a stronger predictor of physician’s evaluation of prognosis than pain diagnosis or severity in chronic pain patients. J Pain.

[29] Risdon A, Eccleston C, Crombez G, McCracken LM (2003). How can we learn to live with pain? a Q-methodological analysis of the diverse understandings of acceptance of chronic pain. Soc Sci Med.

[30] Burns JW, Johnson BJ, Devine J, Mahoney N, Pawl R (1998). Anger management style and the prediction of treatment outcome among male and female chronic pain patients. Behav Res Ther.

[31] Feldman C (2005). Compassion: Listening To The Cries Of The World.

[32] Robak RWN, Poonam R (2011). Psychological needs: a study of what makes life satisfying. N Am J Psychol.

[33] Sprecher S, Fehr B, Zimmerman C (2007). Expectations for mood enhancement as a result of helping: the efects of gender and compassionate love. Sex Roles.

[34] Canevello A, Crocker J (2010). Creating good relationships: responsiveness, relationship quality, and interpersonal goals. J Pers Soc Psychol.

[35] Loizzo JJ, Blackhall LJ, Rapgay L (2009). Tibetan medicine: a complementary science of optimal health. Ann N Y Acad Sci.

[36] Salzberg S (1997). Lovingkindness: The Revolutionary Art Of Happiness.

[37] Boellinghaus I, Jones FW, Hutton J (2012). The role of mindfulness and loving-kindness meditation in cultivating self-compassion and otehr-focused concern in health care professionals. Mindfulness.

[38] Marchand WR (2012). Mindfulness-based stress reduction, mindfulness-based cognitive therapy, and Zen meditation for depression, anxiety, pain, and psychological distress. J Psychiatr Pract.

[39] Marchand WR (2013). Mindfulness meditation practices as adjunctive treatments for psychiatric disorders. Psychiatr Clin North Am.

[40] Bormann JE, Thorp SR, Wetherell JL, Golshan S, Lang AJ (2013). Meditation-based mantram intervention for veterans with posttraumatic stress disorder: A randomized trial. Psychol Trauma Theory Res Pract Policy.

[41] Piet J, Hougaard E (2011). The effect of mindfulness-based cognitive therapy for prevention of relapse in recurrent major depressive disorder: a systematic review and meta-analysis. Clin Psychol Rev.

[42] Carim-Todd L, Mitchell SH, Oken BS (2013). Mind-body practices: an alternative, drug-free treatment for smoking cessation? A systematic review of the literature. Drug Alcohol Depend.

[43] Miller JJ, Fletcher K, Kabat-Zinn J (1995). Three-year follow-up and clinical implications of a mindfulness meditation-based stress reduction intervention in the treatment of anxiety disorders. Gen Hosp Psychiatry.

[44] Carlson LE, Speca M, Patel KD, Goodey E (2004). Mindfulness-based stress reduction in relation to quality of life, mood, symptoms of stress and levels of cortisol, dehydroepiandrosterone sulfate (DHEAS) and melatonin in breast and prostate cancer outpatients. Psychoneuroendocrinology.

[45] Salzberg S (1995). Loving-Kindness.

[46] Neff KD, Beretvas SN (2012). The role of self-compassion in romantic relationships. Self Identity.

[47] Neff KD, Germer CK (2012). A pilot study and randomized controlled trial of the mindful self-compassion program. J Clin Psychol.

[48] Jazaieri H, Jinpa GT, McGonigal K, Rosenberg EL, Finkelstein J, Simon-Thomas E, Cullen M, Doty JR, Gross JJ, Goldin PR (2012). Enhancing compassion: a randomized controlled trial of a compassion cultivation training program. J Happiness Stud.

[49] Gilbert P (2009). The Compassionate Mind: A New Approach to Life’s Challenges.

[50] Gilbert P (2010). Compassion Focused Therapy: Distinctive Features.

[51] Gilbert P, Procter S (2006). Compassionate mind training for people with high shame and self-criticism: overview and pilot study of a group therapy approach. Clin Psychol Psychother.

[52] Laithwaite H, O’Hanlon M, Collins P, Doyle P, Abraham L, Porter S, Gumley A (2009). Recovery After Psychosis (RAP): a compassion focused programme for individuals residing in high security settings. Behav Cogn Psychother.

[53] Kelly AC, Zuroff DC, Shapira LB (2009). Soothing oneself and resisting self-attacks: the treatment of two intrapersonal deficits in depression vulnerability. Cogn Ther Res.

[54] Fredrickson BL, Cohn MA, Coffey KA, Pek J, Finkel SM (2008). Open hearts build lives: positive emotions, induced through loving-kindness meditation, build consequential personal resources. J Pers Soc Psychol.

[55] Hutcherson CA, Seppala EM, Gross JJ (2008). Loving-kindness meditation increases social connectedness. Emotion.

[56] Leiberg S, Klimecki O, Singer T (2011). Short-term compassion training increases prosocial behavior in a newly developed prosocial game. PLoS One.

[57] Pace TW, Negi LT, Adame DD, Cole SP, Sivilli TI, Brown TD, Issa MJ, Raison CL (2009). Effect of compassion meditation on neuroendocrine, innate immune and behavioral responses to psychosocial stress. Psychoneuroendocrinology.

[58] McCracken LM, Vowles KE, Eccleston C (2004). Acceptance of chronic pain component analysis and a revised assessment method. Pain.

[59] Finucane AM, Dima A, Ferreira N, Halvorsen M (2012). Basic emotion profiles in healthy, chronic pain, depressed and PTSD individuals. Clin Psychol Psychother.

[60] McCracken LM (1998). Learning to live with the pain: acceptance of pain predicts adjustment in persons with chronic pain. Pain.

[61] Pilkonis PA, Choi SW, Reise SP, Stover AM, Riley WT, Cella D, Group PC (2011). Item banks for measuring emotional distress from the Patient-Reported Outcomes Measurement Information System (PROMIS(R)): depression, anxiety, and anger. Assessment.

[62] Cella D, Riley W, Stone A, Rothrock N, Reeve B, Yount S, Amtmann D, Bode R, Buysse DJ, Choi SW, Cook KF, DeVellis R, DeWalt D, Fries JF, Gershon R, Hahn E, Pilkonis P, Revicki D, Rose M, Weinfurt K, Hays RD, on behalf of the PROMIS Cooperative Group (2010). Initial item banks and first wave testing of the Patient-Reported Outcomes Measurement Information System (PROMIS) network: 2005–2008. Journal of Clinical Epidemiology.

[63] Jacobson CJ, Farrell JE, Kashikar-Zuck S, Seid M, Verkamp E, Dewitt EM (2013). Disclosure and self-report of emotional, social, and physical health in children and adolescents with chronic pain-a qualitative study of PROMIS pediatric measures. J Pediatr Psychol.

[64] Cleeland CS (1991). Research in cancer pain. What we know and what we need to know. Cancer.

[65] Farrar JT, Young JP, LaMoreaux L, Werth JL, Poole MR (2001). Clinical Importance of changes in chronic pain intensity measured on an 11-point numerical pain rating scale. Pain.

[66] Williams AC, Eccleston C, Morley S (2012). Psychological therapies for the management of chronic pain (excluding headache) in adults. Cochrane Database Syst Rev.

[67] Dworkin RH, Turk DC, Wyrwich KW, Beaton D, Cleeland CS, Farrar JT, Haythornthwaite JA, Jensen MP, Kerns RD, Ader DN, Brandenburg N, Burke LB, Cella D, Chandler J, Cowan P, Dimitrova R, Dionne R, Hertz S, Jadad AR, Katz NP, Kehlet H, Kramer LD, Manning DC, McCormick C, McDermott MP, McQuay HJ, Patel S, Porter L, Quessy S, Rappaport BA (2008). Interpreting the clinical importance of treatment outcomes in chronic pain clinical trials: IMMPACT recommendations. J Pain.

[68] Kabat-Zinn J, Chapman-Waldrop A (1988). Compliance with an outpatient stress reduction program: rates and predicotrs of program completeion. J Behav Med.

[69] Barnhofer T, Chittka T, Nightingale H, Visser C, Crane C (2010). State effects of two forms of meditation on prefrontal EEG asymmetry in previously depressed individuals. Mindfulness.

[70] May CJ, Burgard M, Mena M, Abbasi I, Bernhardt N, Clemens S, Curtis E, Daggett E, Hauch J, Housh K, Janz A, Lindstrum A, Luttropp K, Williamson R (2011). Short-term training in loving-kindness meditation produces a state, but not a trait, alteration of attention. Mindfulness.

[71] May CJ, Weyker JR, Spengel SK, Finkler LJ, Hendrix SE (2012). Tracking longitudinal changes in affect and mindfulness caused by concentration and loving-kindness meditation with hierarchical linear modeling. Mindfulness.

[72] Hofmann SG, Grossman P, Hinton DE (2011). Loving-kindness and compassion meditation: potential for psychological interventions. Clin Psychol Rev.

[73] Boellinghaus I, Jones FW, Hutton J (2014). The role of mindfulness and loving-kindness meditation in cultivating self-compassion and other-focused concern in health care professionals. Mindfulness.

[74] Lutz A, Brefczynski-Lewis J, Johnstone T, Davidson RJ (2008). Regulation of the neural circuitry of emotion by compassion meditation: effects of meditative expertise. PLoS One.

[75] Kelly AC, Zuroff DC, Foa CL (2010). Who benefits from training in self-compassionnate self-regulation? A study of smoking reduction. J Soc Clin Psychol.

